# The masking and making of fieldworkers and data in postcolonial Global Health research contexts

**DOI:** 10.1080/09581596.2019.1609650

**Published:** 2019-06-04

**Authors:** Patricia Kingori, René Gerrets

**Affiliations:** a The Ethox Centre, and Wellcome Centre of Ethics and Humanities, Nuffield Department of Population Health, University of Oxford, Oxford, UK; b Department of Anthropology, University of Amsterdam, Amsterdam, The Netherlands

**Keywords:** Identity, research, management

## Abstract

This paper centres on the roles and contributions of fieldworkers-local data-collectors in Global Health research in postcolonial contexts. It is informed by two separate ethnographies, conducted in two different East African biomedical research institutions. It discusses how common characterisations of fieldworkers as ‘low-skilled’ and ‘local’ make them attractive to research institutions in two important ways – as community-embedded data-collectors thus facilitating community participation and as being unlikely to fabricate data because they lack the skills to avoid detection. This paper questions these assumptions. It draws on Daston’s idea of the ‘scientific persona’ and Fanon’s concepts of mask-making to explore how fieldworkers construct identities and data within their liminal roles. Fieldworkers create particular pseudo-personae or masks for getting and staying employed. They dumb-down CVs and emphasise their similarities with community members in ways which are partially ‘real’ but also ‘fake’. These constructed identities provide fieldworkers with a persona that allows them to fabricate or modify data without raising suspicions. They frequently engage in practices known as ‘genuine fake’ data fabrication which is data perceived as factually correct and verifiable yet methodologically incorrect, hence it is real and fake in varying degrees. We understand the ‘pseudo’ as the blurry space between real and fake where fieldworkers construct their identities and data. Given the seemingly laudable aims of Global Health, we argue that fieldworkers’ masking and making up data signal the need for greater attention by those designing its research, to better understand and address why and how these practices unfold.

## Introduction

…this sounds strange in a way, but sometimes you don’t want the cleverest person [as a fieldworker]… you want somebody who is reliable, to get the good data… from the perspective of a manager… you are looking for …somebody who is… just kind of by the book and gets the data.

(Emily, Senior Researcher, STUDY A)

The idea that science requires anything other than the cleverest mind is strange to many. Yet, it is precisely a perceived lack of higher education that often grants contemporary fieldworkers employment opportunities, in Global Health research, in many postcolonial contexts. This kind of tension is hardly unique to this field; it reflects widespread institutional divisions of labour and hierarchical systems. In this paper, we argue that characterisations of fieldworkers as ‘low-skilled’ and ‘local’ make them attractive to research institutions in two important ways – as community-embedded data-collectors and because they are not deemed clever enough to get away with fabricating data.

Since engagement in labour-related activities is invariably entwined with processes of self-making, this paper also examines how several features of Global Health research can influence said processes of self-making, and, by extension, how Global Health research manifests in practice. Specifically, we explore how a preference in contemporary Global Health research institutions for relatively uneducated, community-embedded fieldworkers in settings where these institutions constitute islands of privilege, and potential opportunity, produces phenomena that we loosely call ‘pseudo Global Health’ (Kingori & Gerrets, 2019). Throughout this paper, the prefix ‘pseudo’ denotes practices operating between ideas of ‘real’ and fake’. While ‘pseudo’ is often used pejoratively we draw on Karl Popper’s conception of pseudoscience and extend it to the phenomena outlined in this paper (1957). Recognising that encounters between research institutions and the people they engage can yield multiple ‘pseudo phenomena’, here we focus on two, both centring on fieldworkers: the construction of particular personae or masks, which are partially authentic, for getting and staying employed, and the production of so-called ‘genuine fake’ data. The concept of ‘genuine fake’ refers to data perceived as factually correct and verifiable yet also methodologically incorrect, hence it is real and fake in varying degrees (Wainer, 2004; Waller, 2013).

Conceptually, we draw on insights from two different theories of masking and personae – Lorraine Daston and Otto Sibum’s (2003) examination of the construction of the scientific persona and Frantz Fanon’s (1986) theory of racial masking. We also make use of David Harvey’s insights regarding neoliberal transformations in labour markets, which concentrate the benefits of labour to groups of ‘core workers’ while easily replaceable, poorly paid ‘peripheral’ workers face shrinking possibilities to surmount the socio-economic inequalities and inequities constraining them (Harvey, 1989; Kasmir & Carbonella, 2008).

Given these tensions, we argue that crafting and wielding masks, and constructing ambiguity between ‘real’ and ‘fake’ – data *and* personae – constitute crucial strategies for fieldworkers when navigating quotidian demands in Global Health research (Crane, 2010). Recalling the words of Emily, quoted above, undertaking medical survey research ‘by the book’ and recruiting fieldworkers who ‘just get the data’ risks obscuring local specificities, thus rendering data collection unresponsive to logistical, practical and ethical needs of fieldworkers and researched populations, while devaluing the skill and craft needed. To manage the expectations of scientists like Emily, fieldworkers develop and use masks to obscure what they consider their real achievements and qualifications – if wielded successfully these masks in turn allows fieldworkers leeway to generate ‘genuine fake’ data that, supposedly better-educated, senior scientists usually cannot differentiate as ‘real’ or ‘fake’.

This paper is informed by two separate ethnographies on fieldworkers, conducted in two different East African biomedical research institutions between 2004 and 2009. Although both ethnographies investigated data collection and its contextual influences in large-scale operations in malaria and HIV research, common themes only became apparent after their completion (c.f. Kingori & Gerrets, 2016). Presenting these findings together allows us to compare and contrast cross-cutting themes. To preserve the anonymity of respondents, the ethnographic examinations are called STUDY A and STUDY B. In both ethnographies, multi-level ethical and scientific approval was obtained. This included not only approval at institutional and national-levels in all relevant countries but also consent from all fieldworkers and researchers involved. In each location, the sensitive issue of data fabrication was discussed with senior scientists, whose feedback has been incorporated into this paper. For example, in STUDY A, senior researchers were keen that the paper reflected their zero-tolerance attitude to data fabrication. The research findings were fed back and verified at each location.

In what follows we bring together insights from postcolonial studies, the history and philosophy of science, anthropology, and sociology. We begin by drawing on historical accounts of masks as techniques in persona construction among bench scientists, before moving to an exploration of similar practices among fieldworkers in contemporary East Africa.

## The construction of the scientific persona

This paper discusses the issue of data fabrication among fieldworkers. Data fabrication is usually discussed in literature among senior or bench scientists. One of the explanations provided for why these scientists fabricate data without being detected is through their construction of credible scientific persona based on ideas of their superior intelligence, cleverness and adherence to a scientific methodology which can act to deflect suspicion of malpractice and bolster their status. Historians of science have used the concept of the scientific persona to examine the relationship between the personal characteristics of ‘the scientist’ and the science they produce (Daston & Sibum, 2003; Shapin, 1989). Invoking Mauss’ understanding of when, why, and where a distinctive ‘scientific persona’ appeared (1938), seemingly authentic science is produced through legitimate techniques, concepts and theories, and by developing and deploying credible scientific identities, epistemological processes and individual characteristics. As Daston and Sibum (2003, p. 2) explain:

Intermediate between the individual biography and the social institutions lies the persona: a cultural identity that simultaneously shapes the individual in body and mind and creates a collective with a shared and recognisable physiognomy…

Becoming an authentic scientist, then, involves developing and displaying multifaceted competence – not only regarding scientific tasks but also with respect to active appropriation and persuasive performance of culturally anchored roles, identities and repertoires to demonstrate reliability, ethically sound values and trustworthiness (Daston & Sibum, 2003). This underscores that constructing and enacting credible scientific personae involves the alignment of substantial effort, skills and experience. While not intending to equate fieldworkers with scientists, this study draws inspiration from the ideas about personae outlined above, because convincingly crafting and performing a credible persona transfers authenticity and authority to scientists (Latour & Woolgar, 1986). Moreover, when successfully wielded, masks can stand in for real skill and authority (Latour & Woolgar, 1986). Hence, a convincingly constructed and performed persona can be viewed as a double-edged sword that can be deployed for ‘good’ – and for ‘ill’. In recent years in discussions of data fabrication, the notion of the successfully enacted scientific personae has been presented as an explanation of how bench scientists have deflected suspicions of scientific malpractice and misconduct (Franzen, Rodder, & Weingart, 2007). This discussion is useful in making visible some of props used by scientists seeking to adopt an authentic scientific persona and some of the techniques they have deployed to evade detection.

In what follows, we apply these ideas to self-making and identity construction in our examination of fieldworkers and their principal activity – collecting data, the raw material for science. Examining how fieldworkers craft and perform a persona reveals how they navigate some of the inequalities and inequities present in contemporary Global Health.

## Historical antecedents to global health fieldworkers

Personae are creatures of historical circumstance; they emerge and disappear within specific contexts… and the concept of persona… can be fruitfully deployed to diverse periods, locales and disciplines…(Daston & Sibum, 2003, p. 3)

Following World War Two, medical research and interventions in Africa increased dramatically in scope and scale, requiring significant numbers of research participants and personnel (Smith, 1967). Graboyes estimates that between 1945 and 1960 ‘more than 200,000 East Africans participated in some form of medical research in Kenya, Tanganyika and Uganda by giving blood, stool, urine or skin samples’ (Graboyes, 2015, p. 8). Though precise figures are lacking, the number of fieldworkers employed in these projects also rose significantly during this period (Browne, 1964).

Reflecting the doctrine of Indirect Rule, the British colonial administration initially relied on local chiefs to advocate biomedical initiatives (Kipkorir, 1980). However, as chiefs were criticised for adhering to traditional beliefs and practices (Kipkorir, 1980) and lost respect among native populations due to their associations with the colonial government (Graboyes, 2015), recruitment began favouring the products of British education and religious systems. Increasingly, Africans recruited for medical research were expected to be ‘young’, ‘educated’ and ‘modern’ (Shadle, 2006).^1^ Familiarity with local culture and language was appreciated but there was little if any preference for fieldworkers hailing from the communities where studies were conducted (Graboyes, 2015). Kipkorir notes that these pressures prompted African employees to quickly learn to wear ‘the mask of a happy worker’ (1980, p. 8). Enabling them to appear compliant or to elude demands by superiors, here, this mask is suggestive of the practices, and challenges, inherent in being a colonial employee. A mask can hide misalignments between intentions and actions, between one’s inner world and one’s projection outward. Thus, following Goffman (1978), convincing masking can help persons successfully navigate diverse social spheres, and their attendant norms and expectations. Moreover, masks can be wielded to project one’s persona and, as will be elaborated below, developing and using masks can also become integral to one’s persona and self-making. Hence the mask, which in Latin means persona (Daston & Sibum, 2003), has theoretical implications for sociology and postcolonial studies (see Fanon, 1986).

The period around independence in Africa saw unprecedented social and employment mobility among East Africans, as colonial administrators prepared to devolve social, educational, economic and political institutions (Green, 2014). In science and research, increased investment in training formerly junior African staff enabled some to be promoted. Africans who had invested in ‘modern’ education expected greater social mobility and better employment (Austen, 2006). This was significant for low-ranking employees in medical research, including fieldworkers, who perceived education as their passport to higher-status and long-term positions.

However, after access to education had expanded during the 1970s, competition for shrinking employment opportunities soared from the 1980s onwards (Tripp, 1997). Amidst this crisis, foreign funding often provided a lifeline for medical research institutions in East Africa. For staff in lower institutional cadres such as fieldworkers, however, features typical of foreign-funded endeavours, such as budget time lines, often meant that employment conditions became insecure and more competitive (e.g., Crane, 2010; Kingori & Gerrets, 2016). With few exceptions, this shift has persisted into the present, and is entangled with the magnified precarity and socio-economic disparities caused by globalisation and economic liberalisation (Harvey, 1989).

## Contemporary ‘community’ fieldworker personae

To examine the personae that contemporary fieldworkers forge and wield, this section highlights four interlocking, influential developments: the rise of ‘community participation’; the lowering of fieldworker status; spatial and social distance within interventions; and fieldworkers as intermediaries between projects and communities.

‘Community participation’ was key to the paradigm shift in international development from earlier ‘top-down’ approaches to attempts at more engagement with researched communities (Green, 2014). Consequently, projects increasingly preferred fieldworkers to be ‘close to’ or in ‘the community’. Furthermore, scholars investigating the institutionalisation of community participation in development interventions observed a concomitant lowering of the status of fieldworkers and communities (Justice, 1986). As casually hired employees, fieldworkers are low ranking in institutional hierarchies.

The pronounced, multifaceted distance – e.g., spatial, social, cultural and linguistic – between expatriate and local staff in contemporary health interventions is another prominent feature. For example, Owen (2010, p. 109) describes how these two categories of NGO staff in resource-poor contexts moved in largely separate social spheres. Importantly, this distance can constrain but also enable. For instance, Wong (2010) found that fieldworkers in Bangladesh and Ghana evaded managerial supervision by emphasising poor infrastructure (such as roads, sanitation or lodging) outside town.

Jointly these accounts point at crucial, often liminal, involvements of fieldworkers as intermediaries between communities and interventions (Turner, 1967). This liminality grants fieldworkers some agency and flexibility in developing personae and acquiring power through their positions as cultural, linguistic and conceptual ‘brokers’ (Bierschenk, Chaveau, & Olivier De Sardan, 2002). Pigg (1992) showed that fieldworkers, whilst being caricatured and misrepresented themselves, also participated in characterising ‘villagers’ as illiterate and difficult; promoting their own brokerage skills by magnifying the distance between senior scientists and villagers. Reinforcing such stereotypes required limited effort, for many development programmes are premised on addressing presumed knowledge deficits among community members (c.f. Brown & Green, 2015).

Insights from the literature hint at fieldworkers’ possible motivations for adopting personae and masking. For Daston and Sibum, masks and masking – as artisanal techniques acquired in the process of becoming an ‘authentic’ scientist – carry mostly positive connotations. Not so for Fanon, who in *Black skin, white masks* (1986) regards them as the causes and consequences of assimilation, racist ideals and Othering in (post)colonial societies that trap mask-bearing blacks in oppressive structures. As Pfeiffer (2003) reminds us, ‘social and racial apartheid’ can accompany Global Health. Following Fanon, one can approach fieldworkers’ mask-making and wielding as an element in their navigations of highly stratified and often inequitable situations, asking whether and how masking enables fieldworkers to escape from the structural conditions ensnaring them.

Thus approached, the masks fieldworkers develop and deploy differ from scientific personae described by Daston and Sibum who speak of masks in positive terms. While masks might enable the disenfranchised wearer, in Fanon’s words, to claim an ‘ethical position in the world’ (1986, p. 214) and thereby some dignity, the bearer gains limited agency because such masks reinforce stereotypes without remedying the structural conditions underlying the inequities, injustice and segregation to which the bearer is subjected. The ways fieldworkers craft and wield masks will be explored further later in the article.

## Genuine fake data

Capturing the extent of data fabrication among fieldworkers was not the aim of our work but studies examining the practice among fieldworkers collecting survey data suggest that the practice is widespread and commonplace (Finn & Ranchhod, 2013). While typically approaching data fabrication as an issue, these studies also suggest that a proportion of the falsified data that fieldworkers generate is less problematic because it is ‘genuine fake’. The notion ‘genuine fake’ data, originating in Demography, has gained traction because it can capture the ambiguity in data that seem accurate and verifiable yet are not collected according to prescribed methodological procedures (Waller, 2013). For example, in a South African study in which data fabrication was detected while fieldwork was still going on, researchers found that the fabrication would not have affected univariate and cross-sectional estimates meaningfully because fieldworkers were able to predict with a significant degree of accuracy survey responses (Finn & Ranchhod, 2013). Although genuine fake data are typically considered a type of fabrication (Kingori & Gerrets, 2016), here we approach them differently, as a lens for analysing particular social dimensions of Global Health research. By exploring *why* fieldworkers produce genuine fake data, and the work and expertise this involves, we aim to address two gaps in the literature on fabrications and scientific misconduct: the limited attention given to fieldworkers (as opposed to scientists); and the side-lining of the skills and expertise that fieldworkers bring to their work, including in the production of ambiguous yet passable data.

Why and how do fieldworkers acquire the ability to create data that stand a chance of passing as real? The phenomenon of genuine fake data provokes questions about knowledge production, and about those who produce it, and as such can generate important insights into Global Health practice, and by extension, we argue, into pseudo Global Health. These questions become salient in locations across East Africa, where nowadays, in contrast to the colonial era, fieldworkers’ presumed familiarity with local conditions, culture, and language are seen as vital for extending the scientific aims of research projects and health-related development interventions to their communities (Green, 2014; Lewis & Mosse, 2006).

However, as the ensuing sections will show, fieldworkers developed and wielded masks and personae as part of their need to navigate working conditions that were often challenging. In such situations, concealing one’s qualifications or exaggerating one’s embeddedness in and affinity for ‘community’ can spell the difference between gaining employment or not. Likewise, acquiring the skills and expertise to produce ‘genuine fake’ data and evade detection can help forge the persona of a competent fieldworker and, perhaps, increase one’s chances of employment. Paradoxically, fieldworkers acknowledged, and sometimes regretted, that genuine fake data did not meet prescribed scientific standards, yet when confronted with working conditions they deemed unfair they also took pride in their experience-based ability to generate empirically grounded data which could dupe highly educated experts.

## Findings

### ‘Talking to their relatives, friends and neighbours’

In both ethnographic research locations, particular assumptions prevailed among senior researchers about fieldworkers’ skills and contributions, and their presumed relations with and access to ‘the community’. We found in both locations that these assumptions were held not only by expatriates but also by some senior African scientists. These assumptions influenced how fieldworkers’ data-collection activities were viewed, and how the fieldworkers were trained and managed. For example, in both locations, epidemiological and economic survey research were recurrent activities for which fieldworkers were recruited and trained. Usually, each survey involved a training workshop, where senior researchers instructed fieldworkers about matters such as interview questions, the collection of faecal samples, or how to make blood smears. Overall, training workshops focused less on the scientific premises of surveys than on issues relevant to conducting them. However, training styles among senior scientists varied substantially. So-called *bosi nzuri –* ‘good bosses’ in KiSwahili, the principal lingua franca in East Africa – generally encouraged interactions, questions and discussions, for instance about survey questions that could be considered offensive by community members, organisational difficulties, and other challenges. In contrast, *bosi mkali* – ‘harsh bosses’ – tended to instruct fieldworkers in an authoritarian fashion, offering little room for dialogue (c.f. Kingori & Gerrets, 2016).

These varying approaches indicate how senior scientists viewed fieldworkers. Some, usually ‘good bosses’, generally valued their contributions, experience and local knowledge. Others treated fieldworkers primarily as implementers of protocols, who merely collected data as stipulated, or viewed them as simply ‘talking to their relatives and friends and neighbours’, as Eugene, an expatriate Senior Researcher, explains:

…we take time to give… [the community] some sense of ownership of the project… and make the most of the tight bond… shared in the community. You know, [fieldworkers] are sons and daughters of the villages [and] when they are working in the field, they… bring the project to the doorsteps, talking to their relatives, friends and neighbours about what we are doing… I think that’s why [the community] really support us.

(In-depth interview, STUDY A)

While senior scientists generally regarded fieldworkers’ kinship relationships, as ‘sons and daughters of the villages’, as an asset, their views differed considerably on the skills and expertise that fieldworkers brought to, or needed for, their work. This paper will explore in-depth how such ideas are examples of the pseudo-personae constructed by fieldworkers which assisted their production of ‘genuine fake’ data.

### ‘We know we have skills’ – fieldworker personae and masking

Fieldworkers’ actual or perceived level of formal education mattered, often profoundly shaping how others viewed and treated them. Fieldworkers Frederick and Floyd voiced a sentiment encountered in both study locations:

Frederick:
There was a time that we were in a certain [annual staff] meeting and somebody just called us… ‘*Mtu wa mkono*’, someone who works with the hands – a labourer. They mean that we are dispensable… Get rid of one of us and there’s hundreds more…

Floyd: Those people who just help.

Frederick: Somebody who has no skill. Somebody just called us that! In a meeting! Where everybody [all the researchers] was [present] …

Floyd: At times it is very difficult, but you take it as it is… you absorb it… But you are a human being…

Ferdinand: You just make it work.

Frederick: Anyway, we know we have skills…

(Focus Group Discussion (FGD), STUDY A)

In daily parlance, *mtu wa mkono*, KiSwahili for menial labourer, referring to casually hired staff, those who mostly follow orders, often carries a derogatory ring – unlike its opposite *mtu wa ubongo*, literally a person with brains, indicating someone educated, on a long-term or permanent employment contract, who tends to give orders. Historically laden and encountered worldwide in numerous permutations, this dichotomy identifies, justifies and reproduces societal inequalities based on actual or alleged educational accomplishments (Buchert, 1994; Willis, 1981).

Moreover, the menial/cerebral opposition can intersect with other unequal dichotomies, reproducing and normalising associated social inequalities (Baumann, 2004; Ortner, 1972). Illustrating such entanglements, during discussions and casual conversations, people in Site B commonly combined the menial/cerebral and educated/uneducated dichotomies with another hierarchical distinction – *watu wa chini* (‘lowly’ people), versus *watu wa kubwa* (‘powerful’ people). After a number of years, as fieldworkers gradually began trusting the authors, they started to reveal that they modified data (c.f. Kingori & Gerrets, 2016). For example, Solomoni, an experienced fieldworker supervisor, said that *watu wa kubwa* seldom adopted his advice or recommendations. Consequently, he made his own alterations to data, which went undetected because he was considered a *mtu wa chini*. Recalling a survey containing a culturally inappropriate, offensive question asking female informants about the frequency of sex with spouses, Solomoni said, his voice resonant with both frustration and resignation:

We just contributed by changing the questions, and our interviewees agree to these [revised] questions. So this is something [scientists] need to think about. But in the end they look down on us. Why do they look down at us? We are doing a big job, we show our contribution, but still they look us down.

(In-depth interview, STUDY B)

Paradoxically, community members often viewed fieldworkers as comparatively well educated, and different from them, because of their employment (Green, 2014). However, working for people with superior educational achievements, fieldworkers were acutely aware of lacking such credentials. While fieldworkers internalised the pervasive dichotomies outlined above, they also developed a persona based on the knowledge and expertise they acquired for their work.

We now turn to how fieldworkers navigate predicaments by strategically crafting and wielding ‘masks’ and personae. When earlier, Frederick said ‘Anyway, we know we have skills’, this was not only the retort of a hurt individual. Encountered in various forms, these allusions – often whispered or delivered as off-hand remarks – hinted at other goings-on. Carefully heeding and following such signals, we probed fieldworkers’ skills and identities; as trust slowly grew between us and them, some began revealing glimpses of what was behind, and at stake in, their masks of the ‘happy worker’ or ‘son or daughter of the village’, which they carefully crafted and deployed. Being respectful was essential, given the sensitivities surrounding the topic but it was also because fieldworkers saw us as markedly different to their superiors which could be observed through where we lived, socialised, and worked.

When discussing the importance of educational credentials, some fieldworkers revealed that they had learned or suspected their certificates and diplomas might render them over-qualified for fieldworker positions. Hence some *understated* qualifications in their CV and job application, to not be too ‘clever’. A carefully tailored CV was an important prop in a fieldworker’s performance and several fieldworkers showed us different CVs they had crafted: one considered ‘genuine’ which listed all their qualifications; and other, abbreviated versions with qualifications matching specific jobs, removing any information that might render them over-qualified and therefore unemployable. Tailoring CVs helped them project personae that approximated institutional expectations, as two fieldworkers explained:

Frederick:
…there are people who can do this work, and they have degrees, like my colleague is saying, but we put them aside… we don’t bring up our certificates.

Interviewer (PK): Why do you do that?

Francis: Because [research institutions] will not accept [it].

Frederick: They won’t accept those diplomas, those degrees because I am a fieldworker

(FGD, STUDY A)

The ‘pseudo personae’ the fieldworkers’ CVs aimed to project were both ‘real’ – for example, regarding their familiarity with the area or regional languages – and ‘fake’ – for example, because of information omitted or manipulated. They explained this increased their chances of employment in Global Health undertakings, which, notwithstanding claims to transparency and merit-based recruitment, often employed relatives (e.g. poorly qualified spouses) or friends of, for instance, expatriate scientists, prominent national scientists, or local staff occupying influential positions. Considered ‘public secrets’ (Taussig, 1999), these were very difficult to challenge, despite belying institutional claims to fair and ‘real’ employment practices based primarily on credentials. These practices diminished trust in organisational procedures and, given precarious employment both in their roles as fieldworkers and in the area in general, fuelled accusations of immorality and insensitivity vis-à-vis staff while bolstering justifications for ‘pseudo practices’ among fieldworkers. Thus, multiple and simultaneous entangled acts of masking and pseudo-practices by senior scientists and fieldworkers occurred alongside each other.

Crafting a convincing mask is one thing, but successfully wielding it is quite another. Moreover, even convincingly worn masks can have drawbacks. Fieldworker Frank had modified his CV so he appeared to be from the study area. However, his superiors, assuming Frank was locally embedded, gave little sympathy or resources to better manage the challenges he encountered:

… if you look at the… drinking water that [fieldworkers] have, you wouldn’t believe it… I’m someone who works, earns a salary and this is the water I drink. The community feels like, ‘For us that’s normal, that’s what we’ve been drinking for the past I don’t know how long.’ … Even for the last one week alone, I’ve had so many diarrhoea cases… my body is, like, what is happening?

(In-depth interview, STUDY A)

Masking as a ‘son of the villages’ put Frank into challenging rural working conditions. Interestingly, community members were complicit in Frank’s pseudo-persona because his identity mattered less than his willingness to facilitate access to resources such as healthcare, food or connections. Conversely, fieldworkers bearing secrets benefited from staying on good terms with community members, who could unmask them, and so were often considered a valuable asset. This multifaceted juggling illustrates how Global Health projects can also serve other – invisible, ulterior – agendas (White, 1993).

Spatial separation between fieldworkers and their superiors facilitated convincing masking. Fieldworkers often were expected, and were sometimes contractually required, to reside in study locations. Visits by senior scientists were typically infrequent and short and they rarely slept ‘in the field’. This distance enabled various senior scientists to dismiss or ignore contextual nuisances or obstacles that fieldworkers encountered, just as it enabled fieldworkers to craft and wield masks and personae that remained convincing for superiors. Acknowledging that their masks brought benefits and burdens, most fieldworkers stressed the importance of understanding and empathising with communities even when they were not well embedded.

Cultivating a credible persona, obviously, has manifold societal relevance. The next section focuses on one strand: crafting and using a credible persona while constructing ‘genuine fake’ data.

## Genuine fakes and (pseudo) personae

In what follows, we show how fieldworkers acquired the ability to generate genuine fake data, a process hinging on their proximity to and knowledge of the field, their social relationships with research participants and other fieldworkers, their distance from their superiors and their ability to wield masks successfully.

In conversations, interviews and observations, it emerged that fieldworkers considered some types of data easier to fabricate than others. For example, data that can be measured or verified relatively easily, such as someone’s height or weight, are more difficult to fabricate than, for instance, blood samples collected among household members selected for malaria testing. Similarly, survey questions can be falsified with varying degrees of ease. Genuine fake data differed from entirely fabricated data in its intention and in the efforts invested in replicating and forging actual responses that research participants might provide. Furthermore, most fieldworkers we spoke to described altering certain questions in a survey rather than fabricating entire surveys, leaving their surveys part genuine and fake. For example, questions about sensitive topics, such as sexual practices, deaths or religious ideas, were most frequently falsified but seemingly innocuous questions about food consumption were also falsified. Asking such questions verbatim risked embarrassing or upsetting informants, a breach of cultural conventions for which the fieldworker would likely be blamed.

It was tacitly understood that fieldworkers would reformulate potentially problematic questions to become answerable by informants, yet how problematic questions would – or should – be altered, and how this might influence answers obtained, was seldom discussed in meaningful detail. However, what senior researchers viewed as leeway for translating or clarifying linguistic nuances, fieldworkers saw as room for interpreting and answering questions on behalf of participants. For example, since food-related survey questions could elicit requests for or expectations of food, and invoke feelings of ethical conflict, guilt and discomfort among informants and fieldworkers alike, fieldworkers sometimes answered questions on behalf of informants, to avoid upsetting them and disrupting community relations:

I never ask those types of questions… I just complete [the questions myself]…because I already know how much these people eat… No-one here eats three meals a day but that’s what I’m meant to ask them. ‘When you are taking [researched drug] were you taking it with a meal?’ That drug is meant to [be] taken three times per day. That means that they are supposed to eat three times a day. So, if I ask them that then they will look at me like, where is the food that I am supposed to be eating with these drugs? There is no food given with these drugs from those people [senior researchers]… So I answer… For the ones with a *shamba* [subsistence farm] I say twice a day and for the ones without I say once… but… I adjust to match harvest times and such things…

(Karen, Fieldworker, In-depth interview, STUDY A)

The data Karen collected were not genuine because she answered for the informant. Yet her answers were not necessarily inaccurate, for she drew on her experience and knowledge when assessing her informant’s living conditions. Indeed, Karen revealed care and consideration in constructing responses, ensuring these closely resembled real-world conditions. Karen’s actions resemble what Umberto Eco called ‘diplomatic forgery’ – forgery that seeks to recover an original that is either lost, hidden or omitted but that the forger believes to exist (Eco, 1994, p. 187). Genuine fake data are based on detailed observations, deduction and inferences, which are arguably key scientific techniques. Fieldworkers often feel that their ‘fake’ data are more ‘genuine’ because they are closer to real-world conditions than, for instance, some questions in surveys. This sense that their falsified data bore a close resemblance to real data has been corroborated by demographic research mentioned earlier in this paper.

Had Karen not been a fieldworker, then she might have had the clout to query or change survey questions, and remove any need for genuine faking. But Karen, and Solomoni in the earlier example, lacked official influence and status. Moreover, their masks prevented them from contributing to scientific discussions about knowledge production. This reinforced impressions that fieldworkers generally were insufficiently clever or cunning to create deviations that could go undetected. Paradoxically, this increased the perceived discretion between written and spoken survey questions that fieldworkers were given. The genuine fake answers Karen recorded, like those of fellow fieldworkers, were grounded in experience and empirical observations, and tailored to minimise the risk of discovery by superiors monitoring data quality and accuracy for malpractice which could be penalised by terminating employment.

Fieldworkers’ actions, acquired and honed in practice, had a pronounced social dimension, although they usually fabricated data on their own. To ensure data quality, scientists built various traps into surveys to detect fabrication, which fieldworkers had to learn how to discover and address. Most fieldworkers we spoke with agreed that this acquired ability to spot traps and generate convincing genuine fake data was what distinguished ‘master data cookers’, as supervisory fieldworker Solomoni described with glee:

…those who are experienced …they say: this questionnaire, this time it differs [from] the others…look at this question, it was not there [before] …these are new questions. If you look, these are the easy questions …when it is a trap question you can know and when it isn’t a trap question, you can know. That discussion is normally done during the break. Yah people discuss! Have you seen those questions? If you answer without care, then the other question will come …to catch you. You see? So you’re supposed to be careful.

(In-depth interview, STUDY B)

Solomoni further explained that ‘master data cookers’ like him screened for similarities and differences between current and previous data-collection protocols when training for a new study. For example, in surveys they looked for potential ‘trick questions’, and those amenable to corner-cutting or fabrication, by utilising their accumulated knowledge and experience. Maintaining their usual outward demeanour, they screened documents in private and selectively shared information gleaned, for instance during workshop breaks or after work, away from superiors. Information-sharing could boost their status but also carried risks, and ‘master data cookers’ carefully guarded their reputations and expertise. Illustrating these social dimensions, fieldworker Shamila, who relished being respected for her expertise in generating fabricated and genuine fake data, recalled admonishing a new colleague that getting away with fabricating data required knowledge, experience and diligence. She recalls explaining to this colleague the importance between fabricating data carefully, in order words producing genuine fake data which could be distinguished from poor quality ‘data cooking,’ which was easily detected:

You just came recently and you’re trying to cook the data? You see now as a result they have just caught you very easily…you’ll be kicked away…Please, do be careful.

(In-depth interview, STUDY B)

Acquired and refined in practice, fieldworkers’ knowledge and skills – epitomised by their ability to generate genuine fake data and circumvent research protocols – were a prominent, though generally tacit, feature in the personae they cultivated. Social and constituted through routine work activities, these personae gave them pride in their expertise, which was essential for making data collection feasible and, only if they cooperated, successful.

## Discussion

In recent decades fieldworkers in East Africa often have been expected to downplay their education and foreground their familiarity with local populations and contexts, in what Le Meur (2006) and Callon and Rabeharisoa (2003) refer to as ‘hyper-localisation’. They argue that staff hailing from ‘the village’ and, more generally, ‘the community’, such as fieldworkers, are treated as being peripheral to global initiatives. Reinforced by fetishising community involvement, this marginalisation fosters the Othering and essentialising of fieldworkers. Such practices support neoliberal transformations in labour markets (Harvey, 1989). Crafting and strategically wielding masks can help fieldworkers navigate these pressures while coping with the ‘industrialisation’ of Global Health, and the considerable social, economic, spatial and linguistic distance between those designing and overseeing studies and interventions and those implementing them.

Paradoxically, the viability of fieldworkers’ masks and masking hinges on embedded assumptions devaluing the contributions of those not directly engaged in ‘big science’. However, there is an interesting difference between our findings and Fanon’s (1986) theorising on masks. In our research, the down-playing of qualifications and appearances of worldliness among some fieldworkers represented an interesting inversion of Fanon’s theory. In our research, we found that fieldworkers actively adopted masks which essentialised and caricatured them as ‘sons and daughters of the villages’ to gain employment and other practical benefits. A well-worn mask could garner other advantages, such as a reduced workload, a modified research protocol or the opportunity to evade managerial control. However, we found in keeping with Fanon that masking contributed to reproducing forms of structural violence. For example, most fieldworkers sought to escape their circumstances by pursuing educational opportunities. This tantalising goal proved elusive for most fieldworkers, who needed to stay close enough to ‘the community’ to retain employment opportunities.

Our findings show that cultivating a credible fieldworker persona intersected with learning how to construct ‘genuine fake’ data because being perceived as not very clever meant that fieldworkers were not considered sufficiently skilled to undertake modifications to protocol without being detected. Hence, one could argue that the conditions that give rise to ‘genuine fake data’ co-constitute ‘pseudo-personae’, and vice-versa. Both are creations by fieldworkers, partially in response to inequalities and inequities attending Global Health research.

While fieldworkers appreciated the employment opportunities offered by Global Health research, its commitment to equity and reducing diseases that disproportionally affect the poor and disadvantaged appeared to be one of several ‘pseudos’ they encountered and navigated, primarily because the often substantial material benefits largely accrued to staff occupying higher positions, for which one usually required higher-level education.

Fieldworkers knew their employment depended on carrying out survey research based on scientific criteria that would be unimplementable in practice without their experience-based modifications. Such mismatches also raise questions about whose work is pseudo, and whose work is better grounded in scientific premises such as empiricism and the verification of findings. From fieldworkers’ perspectives, Global Health research in practice resembles multiple entangled performances, where fake, real and pseudo mingle. In his work, Fanon was keen to emphasise the indignity suffered by the mask-wearer, which could create mental health problems. In this article, we demonstrate certain social and economic consequences in contemporary postcolonial settings for those forced to wear these ‘hyper-localised’ masks – they were paid less, had more precarious contracts and were less able to make contributions to the intellectual life of science.

Substantial scholarship in Global Health has focused on research participants; however, those employed by Global Health have received little attention. Participants and employees are subject to very strict enrolment criteria and particular rules of engagement. We have learnt that research participants regularly circumvent these rules (Fassin, 2007; Kingori, 2015) and from fieldworkers’ accounts we learn that they do too. Given the seemingly laudable aims of Global Health, we argue that these deviations signal where greater attention should be paid by those designing its research and carrying it out, to better understand and address why and how these practices unfold.

## Conclusion

In this paper, we have argued that ethnographic examinations of fieldworkers and the data that they collect provide valuable insights into the blurry space between real and fake in the everyday conduct of Global Health research. The masks and pseudo personae that fieldworkers develop and wield are key to their ability to produce pseudo data.

We have shown that pseudo personae are entangled in numerous ways in the production of genuine fake data. Being seen to be a local community member can gain fieldworkers employment but, in the eyes of various senior scientists, this also diminishes their potential intellectual contributions to science. In response to insufficient formal recognition for their observations, skills or experience, especially when facing challenging or unfair working conditions, or working under ‘bad bosses’, fieldworkers can draw on accumulated, socially acquired skills and experience to alter data-collection tools and responses. Keen to stay employed, they can decide whether or not to answer questions on behalf of respondents as accurately as possible and to produce genuine fake data that stand a good chance of duping superiors into believing these are real.

Although this paper draws on research conducted in postcolonial contexts in East Africa it questions the aim and conduct of Global health initiatives elsewhere. Furthermore, the notion of the pseudo and its interrogation of binary oppositions complicates questions of right/wrong, ethical/unethical, revealing power and structural violence associated with these categories. Destabilising these binaries sheds light on phenomena which usually evade attention and close examination.

## Note

1. This did not make the use of chiefs redundant. Contemporary medical research institutions still employ chiefs, sub-chiefs and village elders in community-engagement strategies, with varying degrees of success and perceived authenticity.
